# Microfluidic Biosensing Systems Using Magnetic Nanoparticles

**DOI:** 10.3390/ijms140918535

**Published:** 2013-09-09

**Authors:** Ioanna Giouroudi, Franz Keplinger

**Affiliations:** Institute of Sensor and Actuator Systems, Vienna University of Technology, Gusshausstrasse 27-29/366-ISS, Vienna 1040, Austria; E-Mail: franz.keplinger@tuwien.ac.at

**Keywords:** biosensors, microfluidic systems, magnetic sensors, magnetic micro- and nanoparticles

## Abstract

In recent years, there has been rapidly growing interest in developing hand held, sensitive and cost-effective on-chip biosensing systems that directly translate the presence of certain bioanalytes (e.g., biomolecules, cells and viruses) into an electronic signal. The impressive and rapid progress in micro- and nanotechnology as well as in biotechnology enables the integration of a variety of analytical functions in a single chip. All necessary sample handling and analysis steps are then performed within the chip. Microfluidic systems for biomedical analysis usually consist of a set of units, which guarantees the manipulation, detection and recognition of bioanalytes in a reliable and flexible manner. Additionally, the use of magnetic fields for performing the aforementioned tasks has been steadily gaining interest. This is because magnetic fields can be well tuned and applied either externally or from a directly integrated solution in the biosensing system. In combination with these applied magnetic fields, magnetic nanoparticles are utilized. Some of the merits of magnetic nanoparticles are the possibility of manipulating them inside microfluidic channels by utilizing high gradient magnetic fields, their detection by integrated magnetic microsensors, and their flexibility due to functionalization by means of surface modification and specific binding. Their multi-functionality is what makes them ideal candidates as the active component in miniaturized on-chip biosensing systems. In this review, focus will be given to the type of biosening systems that use microfluidics in combination with magnetoresistive sensors and detect the presence of bioanalyte tagged with magnetic nanoparticles.

## 1. Introduction

During the last several years magnetoresistive (MR)-based detection methods in combination with microfluidics have received significant research interest in biosensing applications [1,2]. These methods involve the labeling of bioanalyte with magnetic micro- or nanoparticles and the detection of their stray field using integrated MR sensors. Such sensors are commonly used in hard disk drives but due to their characteristics, they emerged as a promising technology for biosensors [3–5]. Specifically, they are compatible with standard silicon IC technology, and thus suitable for integration into a hand held, portable on-chip biosensing system. They possess high sensitivity (having values of saturation field from 0.1 to 10 kA/m and noise level in the range of a few nT/Hz^1/2^). Compared to the superconducting quantum interference device (SQUID)-based ultrasensitive magnetic detection the MR based sensors are operated at room temperature and have low power consumption in the range of 10 mW.

Nevertheless, apart from the MR-based detection methods in biomedical applications, there are several others such as optical detection and fluorescence labeling of bioanalyte [6–10]. The technical challenges for on-chip applications of this method are the size and the cost of the required instrumentation (laser for the excitation of the fluorescent labels and detection optics) and also the photostability with time, narrow excitation range and broad emission spectra of the fluorescent labels. Therefore, using magnetic micro-or nanoparticles as labels is rather advantageous compared to fluorophores because they are stable and thus the measurements can be repeated without a time limitation and without the need of excitation [11,12]. Another advantage of the magnetic labels is that they can be manipulated on-chip by application of a magnetic field gradient. Additionally, the low production cost and the small size of the microfabricated magnetic sensors promote this method for miniaturization and make it well suited for hand held on-chip biosensing systems.

In order to understand the working principles of the existing microfluidic biosensing systems which use magnetic micro- or nanoparticles and MR-based sensors it is essential to first understand the fundamentals of the involved phenomena. Hence, the following four sub-sections will focus on explaining the phenomenon of superparamagnetism, the MR effect and its application in highly sensitive sensing technologies.

### 1.1. Superparamagnetism

Superparamagnetism is a phenomenon that appears in ferromagnetic or ferrimagnetic nanoparticles. Particles below the Curie or Néel temperature with linear dimensions of approximately 1–20 nm or less will consist of a single magnetic domain, which means that the particles will be in a state of uniform magnetization at any field [1,2,13]. Due to their small size, they would need higher energy to divide themselves into magnetic domains than the energy needed to remain as a single magnetic domain. The total magnetic moment of a nanoparticle is comprised of all the individual magnetic moments of the atoms that form the nanoparticle.

Due to the magnetic anisotropy, the magnetic moment of the nanoparticle usually has two stable orientations (which define the easy axis of the nanoparticle) and is separated by an energy barrier. This barrier is proportional to the volume of the particle. At any finite temperature *T*, the total magnetic moment of the nanoparticle will fluctuate with a finite probability that the moment will flip from one easy direction to another. The mean time between two flips is called the thermal relaxation time *τ* and is given by Equation (1):

(1) $$\tau =\tau_{0}e\frac{K_{u}V}{kT}$$

where *kT* is the thermal energy barrier at temperature *T* (*k* is the Boltzmann constant) and *K_u_V* is the energy barrier for magnetization reversal of a magnetic particle of uniaxial anisotropy *K_u_* and volume *V*.

The phenomenon of superparamagnetism is observed when a particle is small enough so that *τ* is very small compared to the time-scale of the experiment to which it is exposed. In particular, an assembly of ferromagnetic particles behaves as superparamagnetic if the particles are single domain and when the thermal energy at the temperature of the experiment is sufficient to equilibrate the magnetization of the assembly in a time shorter compared to that of the experiment. The particles behave similar to paramagnetic following the Langévin model Equation (2):

(2) $$M=Nm(\text{coth }a-\frac{1}{a})$$

where *m* is the magnetic moment of the particle, *N* the number of magnetic moments per unit volume and $a=\frac{\mu_{0}mH}{K_{\text{B}}T}$ is the ratio of the two energy terms.

For small particles at room temperature the energy barrier becomes comparable to the thermal energy *kT*; as the size of the particle decreases the magnetic anisotropy energy per particle, which is responsible for holding the magnetic moment along certain directions, becomes comparable to the thermal energy. Under these conditions, the thermal fluctuations induce random flipping of the magnetic moment with time. Consequently, the particles behave as superparamagnetic. The difference compared to paramagnetic materials is that superparamagnetic particles can align and reach saturation at relatively low fields and their magnetization can be much greater than that of ordinary paramagnetic materials [2].

Additionally, energy absorption due to Néel relaxation occurs in such nanoparticles. When an external alternating magnetic field is applied, the magnetic dipole moments of the nanoparticles will be rapidly reoriented. This depends on the frequency and strength of the applied field, the temperature and the size of the nanoparticles.

Superparamagnetism is experimentally observed based on two requirements; first, the magnetization curve exhibits no hysteresis and thus no remanent magnetization *M*_r_ is present and second the magnetization curves at different temperatures must superimpose when plotted against *H/T*. Imperfect *H/T* superposition can result from a broad distribution of particle sizes, changes in the spontaneous magnetization of the particle as function of temperature, or anisotropy effects [1,13].

### 1.2. Magnetic Nanoparticles

In general, the magnetic behavior of nanoparticles strongly depends on their dimensions [2]. As the particle size is reduced towards a minimum critical size, approximately below 500 nm, domain wall formation becomes energetically unfavorable. Magnetization changes do not occur through domain wall motion anymore but rather through the rotation of spins. As the particle size is decreased further, anisotropy energy, which stabilizes the orientation of the total magnetic moment in the nanoparticle, can become comparable to the thermal energy. In this case, if the observation time is longer compared to the thermal relaxation time (as shown from Equation 1) the total magnetic moment will fluctuate and the particle behaves as a superparamagnetic one (MNPs).

Such nanoparticles are of great interest in biomedical research. Throughout the years they have facilitated laboratory diagnostics, DNA sequencing, cell analysis, medical drug targeting and have served as contrast agent for magnetic resonance imaging (MRI), for tumor therapy or cardiovascular disease [11]. One of the main advantages of such nanoparticles is their controllable size, which ranges from a few nanometers up to hundreds of nanometers. This makes them very attractive for coupling with biological entities (e.g., viruses, bacteria, cells, genes *etc.*), which have comparable sizes [14]. Once their surface is coated with bioactive ligands they can bind and interact with biological entities thus providing a key method of labeling or addressing these entities. Another major advantage is their magnetic nature; they can be controlled and manipulated by an external magnetic field gradient [15,16]. The recent developments in nanotechnology and biotechnology enabled this modulation and tailoring of their composition, size, surface functionalization and magnetic properties.

For their biomedical applications, the MNPs should be suspended in an appropriate carrier liquid, forming magnetic liquids, which are called ferrofluids. The most commonly used magnetic materials are iron oxides (e.g., γ-Fe_2_O_3_ or Fe_3_O_4_) and the carrier liquids are water or various oils [1]. Due to their small size, the MNPs suspended in liquids usually do not agglomerate due to magnetic dipole interaction. Nevertheless, for a stable suspension the MNPs must be protected against agglomeration due to van der Waals interactions [17]. This can be achieved by appropriate surface modification, which also acts as a functionalization layer for further conjugation with bioactive molecules or targeting ligands. In particular, MNPs can be functionalized either with organic materials (e.g., polymers) [17,18], inorganic metallic materials such as gold [19] or oxide materials (e.g., silica) [20].

Finally, the biomedical applications of MNPs impose strict requirements on their physical, chemical and pharmacological properties e.g., chemical composition, granulometric uniformity, crystal structure, magnetic behavior, surface structure, adsorption properties, solubility and low own toxicity [21]. In order to fulfill these requirements several fabrication methods are being used and some details and extended reviews on MNPs can be found in [14,21–26].

The detection of magnetic particles in static fluids or under fluidic flow requires sensors with high sensitivity to magnetic fields in the range of 1 mT and below [1,3–5]. The following chapter presents the working principle of such highly sensitive magnetic sensors, which are used in biosensing systems for the detection of magnetic micro- and nanoparticles.

### 1.3. Magnetoresistance Sensors

Magnetoresistance (MR) is the effect during which the electrical resistance of a material changes when an external magnetic field is applied to it. It was first discovered in the 1850s but it was the progress of modern microelectronics and solid-state technology in the late 20th century, which enabled its broad applications in industrial sensors with high sensitivity and wide dynamic range as well as in data storage devices [1,27].

The basic principle of the MR effect is described by Equation (3):

(3) R=f(B)

as being the variation of a material’s resistance *R* as a function of the externally applied magnetic field *B*. Due to the Lorentz force, it is expected that the application of a magnetic field to a region which contains moving electrons will cause a change in their trajectories, yielding a change of the effective resistance of the medium containing the electrons [27]. It should be mentioned that the change in resistance is expected to be different for a current flowing parallel to the field from that of a current flowing across the field. The usual figure of merit for magnetoresistance is the MR ratio commonly defined by Equation (4):

(4) $$MR\%=\frac{R_{\left(H=H_{sat}\right)}-R_{\left(H=0\right)}}{R_{\left(H=0\right)}}$$

and indicates the maximum signal that can be obtained from the sensor.

The most interesting feature of MR sensors for biomedical applications is their ability to detect very weak magnetic fields (nT) at room temperature. A great number of research groups worldwide is focusing on the design and development of magnetoresistive biochips used for biomolecular recognition and aiming for the detection of single molecule interaction [28–35]. These applications are based on the giant magnetoresistance (GMR) effect (GMR multilayers and spin valves) and the tunneling magnetoresistance (TMR) effect [36,37].

#### 1.3.1. Giant Magnetoresistance Sensors

Similar to other MR effects, GMR is the change in electrical resistance in response to an applied magnetic field. It is a quantum mechanical effect based on spin dependent scattering in magnetic multilayers. Sensors based on this effect have received increasing interest for biomedical applications because they have been proven to exhibit higher sensitivity at low fields than other MR based sensors, thus being more suitable for the detection of biological target species by means of immunomagnetic assays in combination with MNPs [1,33,35,38–41]. GMR structures have an advantage in size, power consumption, cost and thermal stability with respect to search coil, fluxgate, SQUID, Hall and spin resonance sensors. Furthermore, GMR sensors are also ideal for low cost applications since they are easily energized by applying a constant current and the output voltage is a measure of the magnetic field [33].

**Figure 1:**
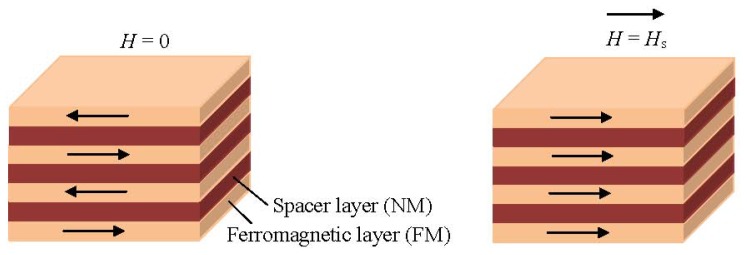
A typical giant magnetoresistance (GMR) structure.

In principle, a typical GMR structure consists of a pair of ferromagnetic thin film layers separated by a non-magnetic conducting layer. The change in the resistance of this multilayer arises when the externally applied magnetic field aligns the magnetic moments of the successive magnetic layers as shown schematically in Figure 1.

**Figure 2:**
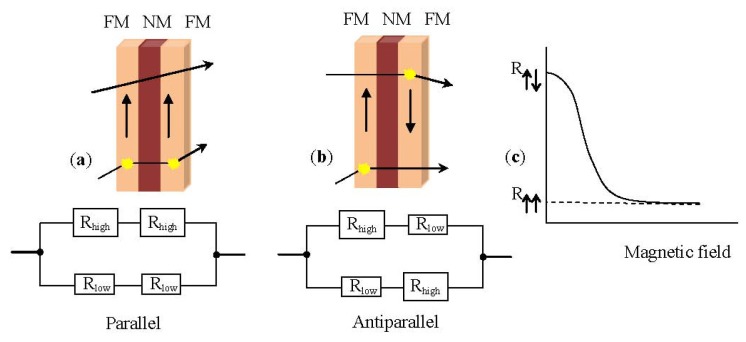
(**a**) Parallel arrangement of a trilayer structure, consisting of a pair of ferromagnetic layers (FM) separated by a non-magnetic layer (NM) with the equivalent resistor network; (**b**) Antiparallel arrangement of the same trilayer structure with the equivalent resistor network; (**c**) Graphic illustration of the resistance variation as a function of the applied magnetic field.

In the absence of a magnetic field, the magnetic moments of the magnetic layers are antiparallel. Once a magnetic field is applied, the magnetic moments of the magnetic layers align with respect to each other; their magnetizations are parallel. This yields a drop in the electrical resistance of the multilayer. Basically, the GMR is derived from the interaction of current carrying electrons and the magnetization of the host magnetic material. In the presence of a magnetic field the spin-dependent electron scattering within the structure reduces and the electrical resistance decreases [1,31]. Figure 2 shows a resistor network used to model the simplest GMR structure, a trilayer. The spin-up and spin-down electrons are represented by two parallel circuits and the resistance of the different layers represented by resistors is given by Equations (5) and (6):

(5) $$R_{P}=\frac{R_{↑↑}\;R_{↑↓}}{R_{↑↑}+R_{↑↓}}$$

(6) $$R_{\text{AP}}=\frac{R_{↑↑}+R_{↑↓}}{2}$$

For the GMR effect in a magnetic multilayer structure to occur, one should be able to orient the magnetic moments of the magnetic layers parallel to each other by application of a magnetic field and antiparallel when the field is zero. This antiparallel alignment of the magnetization (Figure 2b) is accomplished by the interlayer exchange coupling [4,5]. This coupling is mediated by the mobile electrons in the non-magnetic (NM) layer similarly to Ruderman-Kittel-Kausya-Yosida (RKKY) interaction between localized magnetic moments present in a matrix of non-magnetic metal. This interlayer exchange coupling oscillates between ferro- and antiferromagnetic as a function of the thickness of the NM layer. Careful tuning of the thickness of the NM layer during fabrication leads to the desired antiparallel magnetization alignment at zero field.

#### 1.3.2. Spin Valve Sensors

**Figure 3:**
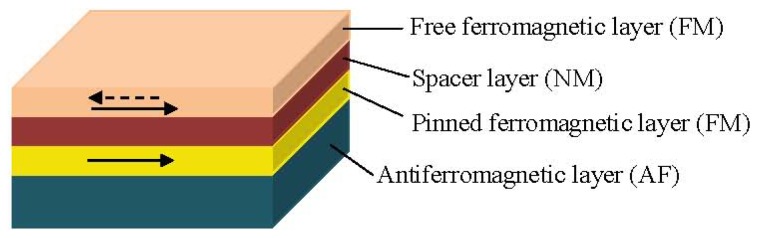
Typical spin valve structure.

Another method to change the alignment of the magnetic moments in the magnetic layers is by tuning the coercivity of one of the FM layers to a higher value. This is achieved at the spin valve structures, which are also based on the GMR effect [1,27,28,42]. Specifically, one FM layer is pinned by the exchange coupling with an adjacent antiferromagnetic layer (see Figure 3) and the other unpinned FM layer is free to rotate with the externally applied field. This way, a parallel or antiparallel magnetization alignment is achieved [43]. Typically, a spin valve structure consists of four metallic layers; an antiferromagnetic pinning layer, a pinned magnetic layer, with a fixed magnetization by an exchange coupling field from the antiferromagnet, a conducting spacing layer (e.g., Cu) and a free magnetic layer whose magnetization rotates according to the signal field as shown in Figure 3 [1,28].

In a magnetic layer the free electrons align their spins to the orientation of the magnetic moment in the layer. Once a potential occurs across the spin valve, the spin-polarized electrons retain their spin alignment as they move through the structure. When these electrons encounter a material with a magnetization pointing in the opposite direction, they need to flip spins to find an empty energy state in the new material. This flip requires extra energy, which causes the structure to have a higher resistance than when the magnetic materials are polarized in the same direction. Thus, the electrical resistance of the spin valve structure is low when the magnetizations from the pinned layer and the free layer are parallel and high when they are antiparallel [28].

#### 1.3.3. Tunneling Magnetoresistance Sensors

**Figure 4:**
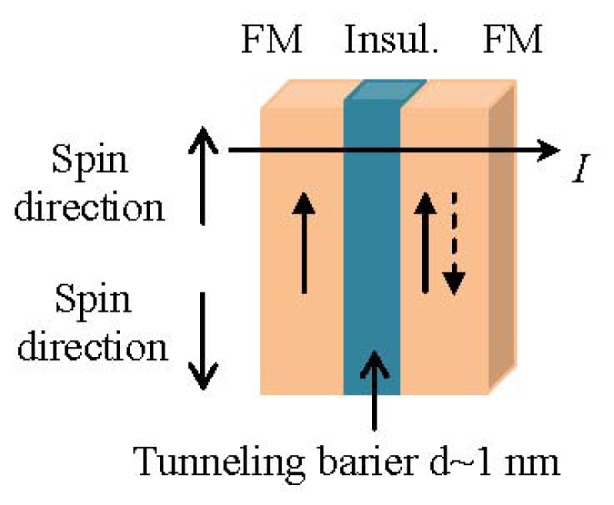
Spin up electrons of a trilayer structure consisting of a pair of ferromagnetic layers (FM) separated by an insulating layer.

The tunneling MR effect (TMR) is a quantum mechanical phenomenon and it occurs in magnetic tunnel junctions (MTJ) which are structures consisting of two ferromagnetic layers separated by a very thin insulating layer [44]. In short, if the insulating barrier, which is between the two ferromagnetic electrodes, is thin enough (in the order of a few nanometers), the electrons can tunnel quantum mechanically through the insulating barrier; a tunnel current flows between the ferromagnetic layers (see Figure 4).

In the case of parallel magnetizations (ferromagnetic coupling), the electrons will tunnel through the insulating layer; since spin is conserved in the tunneling process, the current is larger for parallel than for antiparallel magnetizations. In the case of antiferromagnetic coupling there is a low tunneling probability. As a result, an MTJ can operate as a switch between a low state and a high state of electrical resistance.

It has been proven by several research groups that MTJ sensors reach much higher MR ratios at room temperature in comparison to GMR and spin valve sensors [45–47]. Therefore, the application of MTJs could be preferable than spin valve sensors for the achievement of an extremely sensitive detection.

Nevertheless, in all types of MR thin film sensors an interaction, which couples the FM layers antiferromagnetically across the NM spacer, has to be present.

### 1.4. Microfluidics

Many chemical, biological, and biophysical processes and experiments take place in liquid environments. The chips used during these processes are called microfluidic devices. Microfluidics is also often defined as the technology that moves nanoscale fluid volumes inside channels of micrometer size [48,49]. Microfluidics provide a rapidly growing platform for developing new systems and technologies for an ever-growing list of applications in biotechnology, life sciences, public health, pharmaceuticals and agriculture [50–52]. In this article, focus will be given on their biomedical applications such as point-of-care diagnostics and cell sorting, manipulation and analysis in combination with magnetic methods [49]. Some of the advantages of microfluidics in such applications are the small sample volumes required for testing which leads to greater efficiency, parallel processing of samples and thus fast sampling times, accurate and precise control of samples as well as diverse integration of different detection methods yielding greater sensitivity and low power consumption and low production costs thereby permitting disposability.

## 2. Microfluidic Biosensing Systems: State of the Art

A biosensor is a device used for the detection of bioanalyte consisting of a biological recognition component, which interacts or reacts with the bioanalyte under study, and a transducer which converts this recognition component into a measurable electric output signal. Several ultrasensitive MP-based biosensors suitable for early clinical diagnostics have been reported recently [36,53–55]. Magnetic biosensors rely on the detection of either the binding of functionalized MNPs to a functionalized magnetic sensor surface or the trapping of the MNPs in the vicinity of this sensor.

In practice, what is actually detected by the magnetic sensor is the magnetic stray field of the magnetically labeled bioanalyte when trapped in the vicinity of the sensor or when interacting with a complementary bioanalyte bound to the surface of the sensor. For example, if we consider a magnetic biosensor for protein assay its operation is similar to the widely used diagnostic tool ELISA (enzyme-linked immunosorbent assay) [56–59]. In the ELISA test an antibody is immobilized on the surface of the sensor and the bioanalyte of interest selectively binds to this antibody. The assay is completed by introducing a detection antibody labeled with a fluorescent molecule tag. On the contrary, in magnetic biosensors a magnetic tag (the MNP) is used [60]. As mentioned above, the magnetic sensor detects the stray field from the magnetic tag to derive the number of captured bioanalyte.

Researchers from Stanford University presented in [31] a GMR biochip for Human Papillomavirus (HPV) genotyping and it was one of the first reports, which proved the detection of magnetic particles with attached bioanalytes or the detection of synthetic analytes in solutions with impurities. The GMR sensor used for these experiments had a bottom spin valve structure: Si/Ta(5)/seed layer/IrMn(8)/CoFe(2)/Ru/(0.8)/CoFe(2)/Cu(2.3)/CoFe(1.5)/Ta(3), all numbers in parenthesis are in nanometers. Each chip had 32 pairs of GMR sensors, which were connected to the bonding pads on the peripheral by a 300 nm thick Ta/Au/Ta lead. Each sensor consisted of 32 spin valve strips connected in series and additional fabrication details can be found [3]. In the absence of an applied field, the total resistance of one sensor was around 35 kOhms. The magnetoresistive ratio was 12% after patterning. The pinning direction of the spin valve was in-plane perpendicular to the sensor strip. The easy axis of the free layer was set by the shape anisotropy to be parallel with the sensor strip. This configuration allowed the GMR sensors to work at the most sensitive region of their MR transfer curves. The researchers performed their experiment in two phases: first, the repeated detection of one HPV sample to test the consistency of the GMR sensor and second, the detection of a set of different HPV samples to demonstrate the GMR sensor’s reliability. Their GMR sensors showed an accuracy of ~90%, with good signal consistency across chips and they also showed that theoretically it is feasible to detect very small concentrations (<1 pM) of DNA targets by deploying a right combination of GMR sensors and MNP labels. Later in [61] the same group showed that magnetically responsive nanosensors that have been scaled to over 100,000 sensors per cm^2^ could be used to measure the binding kinetics of various proteins with high spatial and temporal resolution. The researchers reported that they could detect as few as 0.6 particles per μm^2^ with their GMR sensor arrays.

MagArray Inc. [62], founded in 2005, successfully commercializes the magneto-nano sensor technology developed at Stanford under Federal funding.

The authors of [63] recently reported a quick, sensitive genotyping method for human hepatitis B virus (HBV) based on a specially designed GMR biochip, which they combined with magnetic nanoclusters (MNCs), PCR and line probe assay. The GMR multilayers were deposited by DC magnetron sputtering onto Si wafers with a structure of NiFeCo (6 nm)/[Cu (2.2 nm)/NiFeCo (1.5 nm)]10/Ta (100 nm). The sensors based on the GMR multilayers were fabricated by lithography, ion beam etching and lift-off technology [64]. Each chip had eight GMR sensors for detection, including three different probes groups, one blank control group (NTC), and four reference sensors. On top of the sensor, a 300 μm in width and 500 μm in depth SU8 microchannel was formed by photo lithography. Finally, a polydimethylsiloxane (PDMS) cover of 2 mm in thickness was adopted to encapsulate the microchannel, which was used to inject the samples into the sensor detection area. The advantage of this method is that very little sample is used for multi-biomarker detection, and after examination, the sample can be collected. When no MNCs were on the sensor’s surface, the resistance change of the GMR sensor at this time was considered to be a reference signal. When nanoclusters were settled on the surfaces of the sensors, a vertical magnetic field of 18.4 kA/m was applied. However, even after hybridization, the detection time of the GMR sensor is only 15 min, which is much lower than the traditional 2 h. Nonetheless, the surface of the GMR sensor has to be specifically modified prior to hybridization which risks aging and contamination of the modification layer.

In 2010 [65], scientists at Philips developed the Magnotech, a new biosensor platform, which uses MNPs to concentrate, separate and detect target molecules. According to Philips, this type of technology enables blood testing in a more patient friendly manner providing results in a matter of minutes at the point-of-care [66]. One of the greatest advantages of Philips’ new biosensor is that it uses a disposable cartridge that automatically fills itself from a single drop of blood and once filled no additional fluid movement is necessary. The entire assay process within the cartridge is performed by externally applying magnetic fields through electromagnets to control the movement of the functionalized MNPs, which are already in the cartridge (preloaded during manufacture). The detection of the number of bound MNPs follows using an optical technique based on frustrated total internal reflection. Specifically, light hitting the underside of the sensor’s active surface is normally reflected without any loss in intensity (total internal reflection) if illuminated at the correct angle, However, when MNPs are bound to the opposite side of the surface they scatter and absorb the light thus reducing the intensity of the reflected beam. These intensity variations in the reflected beam, which correspond to the number of bound MNPs, are detected by a CMOS image sensor similar to that used in a digital camera.

Another research institute that developed such a magnetic biosensor is Imego [67]. The Imego researchers designed a sensor system based on MNPs with a modified surface to bind virtually any specific molecule to be detected. According to Imego, upon binding of target molecules (e.g., antibodies) to the MNPs, the hydrodynamic particle volume increases, *i.e.*, the more target molecules that get stuck on the MNPs, the larger the particles will grow. This increase in particle size can be determined using dynamic magnetic measurements in a procedure developed and patented by Imego [68]. They also developed the DynoMag^®^ system, an instrument capable to measure the frequency dependent magnetic susceptibility of nano particle systems and find experimentally a good correlation between antibody concentration in a sample and the observed change in hydrodynamic volume of antibody binding MNPs [69]. This AC susceptometer has a frequency range from 1 Hz up to 200 kHz with a resolution in magnetic moment of 3 × 10^−11^ Am^2^ or in volume susceptibility 4 × 10^−7^ (SI-units) at 1 kHz and excitation amplitude of 0.5 mT.

The authors of [70,71] presented a few years ago a low-power scalable frequency shift MNP biosensor array in bulk CMOS, which provides single bead detection sensitivity without any (electrical or permanent) external magnets, which is the main advantage of this device. The sensing method is based on high stability integrated oscillators with on-chip LC resonators. An AC electrical current through the on-chip inductor generates a magnetic field and polarizes the MNPs present in its vicinity. This increases the total stored magnetic energy in the space and hence the effective inductance of the inductor. Due to the increase in inductance a down-shift in the oscillation frequency occurs. Therefore, with this method, no external magnetic field biasing is needed and the sensing system can be completely implemented in a planar process, such as CMOS. In order to prove the concept, a low-cost polydimethylsiloxane (PDMS) microfluidic structure was fabricated and bonded to the CMOS sensor chip. A single magnetic bead of 2.4 μm diameter resulted in a frequency shift of 2.6 ppm, which was easily detected (for an oscillation frequency *f*_0_ = 928 MHz). In a separate experiment, a single carboxylic magnetic bead of 1 μm diameter, at the same oscillation frequency as before, resulted in a frequency shift of 0.25 ppm, still detectable by the system.

However, most of the research mentioned above has been performed either in static liquid, by pipetting MNPs directly onto the magnetic sensors and allowing them to settle, or by transporting reagents and MNPs to the sensors in microfluidic flow and allowing them to chemically bind. These methods generally employ a “sandwich” approach to immobilize bioanalytes [72–74]. First of all, one microbiological substance, the “probe”, is immobilized on top of a magnetic sensor. Secondly, the bioanalyte, also called the “target”, passes by the probes. Targets and probes bind specifically, via e.g., antigen-antibody or ligand-receptor interaction. Finally, MNPs, which can react with targets, are introduced to the sensor’s surface to let them bind with the targets. The biggest disadvantage of this type of biosensors, is that they are prone to time-dependent changes of the functionalization layer such as aging and contamination. Long-term stability is therefore an issue with those types of biosensors. It is also difficult to control or acquire the amount of immobilized probes.

**Figure 5:**
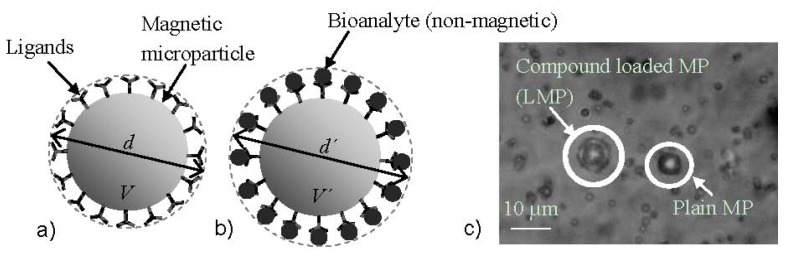
(**a**) Magnetic microparticle coated with ligands having an aggregate diameter *d* and a volume *V* and (**b**) Magnetic microparticle coated with ligands and attached bioanalyte of total diameter *d*′ and total volume *V*′; (**c**) Micrograph of the plain magnetic microparticles with a diameter *d* of app. 6.2 μm (MP), and the same streptavidin coated, magnetic microparticles attached to biotin coated, polystyrene microparticles (of app. 0.98 μm diameter *d*_p_) with a total diameter *d*′ of app. 8.2 μm (LMPs) [32].

Researchers from the Vienna University of Technology in cooperation with the King Abdullah University of Science and Technology introduced a method utilizing microfludics and MNPs which does not rely on functionalization of the sensor surface [32,75,76]. This biosensing system utilizes the innovative concept of the velocity dependence of MNPs due to their volumetric change when bioanalyte is attached to their surface via antibody-antigen binding (see Figure 5). When the MNPs are attracted by a magnetic field within a microfluidic channel, their velocity depends on the presence of bioanalyte. Specifically, their velocity decreases significantly when the MNPs are covered by (nonmagnetic) bioanalyte due to the increased drag force in the opposite direction to that of the magnetic force. The compounds that are formed when bioanalyte covers the surface of the MNPs are called Loaded MNPs (LMNPs).

**Figure 6:**
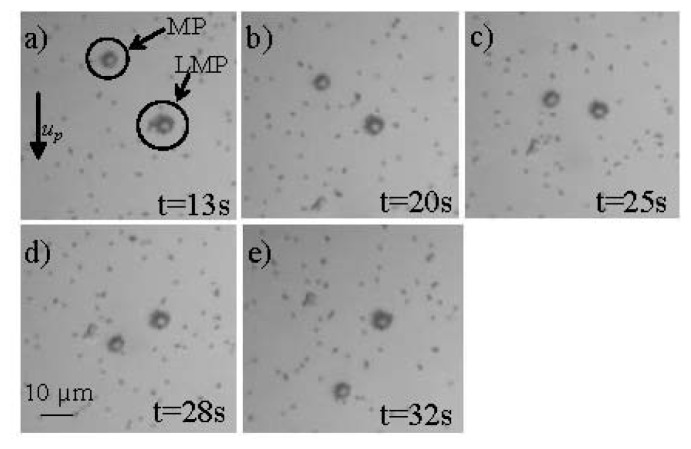
Optical microscope images of a typical measurement of the movement of the plain MPs and the LMPs (described in Figure 6) inside the microfluidic channel (*u*_p_ velocity of MPs) when accelerated by an external magnetic field at (**a**) 13 s; (**b**) 20 s; (**c**) 25 s; (**d**) 28 s; (**e**) 32 s [32].

The magnetic force *F*_ma_*_g_* acting on a single MNP, approximated by a point-like magnetic dipole with a magnetic moment m is proportional to the magnetic flux density *B*. In the case of a superparamagnetic MNP this force is given by Equation (7), assuming the magnetic susceptibility of the surrounding medium is zero:

(7) $$F_{\text{mag}}=\frac{V\chi }{2\mu_{0}}\nabla B^{2}$$

where V is the volume of the MNP, χ is the susceptibility of the MNP and μ_0_ is the permeability in vacuum. The hydrodynamic drag force acting on the MP is proportional to the radius of the microparticle and a consequence of the velocity difference Δ*u* between the MNP and the fluid. In the present case, the fluid is static therefore Δ*u* is equal to the velocity of the MNP. For a spherical particle with radius *r* it is:

(8) $$F_{\text{d}}=6\pi \eta ru$$

where *η* is the viscocity of the surrounding sample fluid. By equalizing Equations (7) and (8), one can determine the particle velocity that can be generated by a magnetic force in a surrounding static fluid. Bioanalyte attaching to the MNP will form an enclosing layer (LMNP) and increase the radius to *r*′ ≈ *r* + 2*r*_p_, where *r*_p_ is the radius of the bioanalyte (see Figure 6a,b). This increase will only have an influence on Equation (8) and thus the velocity will be given by:

(9) $$u_{\text{p}}=\frac{r^{3}\chi }{9\mu_{0}\eta r^{\prime }}\nabla B^{2}$$

Experiments showed a promising 52% decrease in the velocity of the LMNPs in comparison to that of the MNPs when both of them were accelerated inside a microfluidic channel using an external permanent magnet (see Figure 6). The microfluidic device that was utilized for the experiments consisted of two sandwiched calcium-fluoride (CaF_2_) wafers, each one with a thickness of 1 mm. The microfluidic channel was fabricated from SU-8 in between the CaF_2_ wafers with a volume of approximately 0.2 μL [32].

The researchers are currently working on achieving the motion of the MNPs through sequentially actuated, silver conductive microstructures, which are integrated underneath a microfluidic channel and are controlled by a programmable microprocessor [77,78]. The silver microstructures consisted of 18 conductors having a 10 μm width and a distance of 8 μm between them. The microfluidic channel was fabricated using standard photolithography process and a dry photoresist thin film (Ordyl SY355) of 55 μm thickness. 2 μL of MNPs (Micromod) coated with carboxylic acid having different diameters (2 μm–6 μm) with a diluted concentration of 1 mg/mL were injected to the microfluidic measurement channel. A DC current of 50 mA was applied sequentially to each conductor, controlled by the programmable microprocessor. It was proven that by sequentially applying the current to the different conducting elements, the MNPs were moved from the right to the left conductors. At the moment, the movement of the MNPs is captured by a Samsung VP-HMX20C camcorder mounted on a Carl Zeiss Microscope and a digital image processing method. However, the group is actively investigating the integration of GMR sensors to detect the motion of MNPs [79,80].

In results presented in [79] spin valve GMR sensing elements were deposited using a magnetron sputtering system on a Si/SiO_2_ substrate with the following bottom pinned structure: Substrate/MgO 15/Ni_80_Fe_20_ 2.5/Ir_17_Mn_83_ 9/Co_50_Fe_50_ 4.5/Ru 0.8/Co_50_Fe_50_ 1.0/Ni_80_Fe_20_ 4/Co_50_Fe_50_ 1.5/Cu 4/Co_50_Fe_50_ 0.8/Ni_80_Fe_20_ 5/Ru 1 (all thicknesses in nanometers) [76]. The GMR sensor had four GMR sensing elements with two active and two reference sensors in a Wheatstone bridge configuration. A magnetic microactuator (MMA) consisting of conducting loops that produced magnetic field gradients and thus exerted a force on the MNPs was then fabricated on top of the GMR sensor. Finally, a microfluidic channel using the same technology as in [78] was fabricated on top of the MMA. The maximum MR was approximately 2% and the average sensitivity in the linear region was approximately 0.5%/mT without application of an external bias field. MNPs Dynabeads M270 coated with carboxylic acid of 2 μm diameter were successfully detected by the sensor.

The same groups introduced recently another novel biosensing method [34,81], which again does not rely on functionalization of the sensor surface. Current carrying microstructures in combination with mechanical microtraps are used to immobilize MNPs. Bioanalyte detection is based on the difference in size between bare MNPs and MNPs with attached bioanalyte, which causes a different number of particles to be captured in the microtraps.

**Figure 7:**
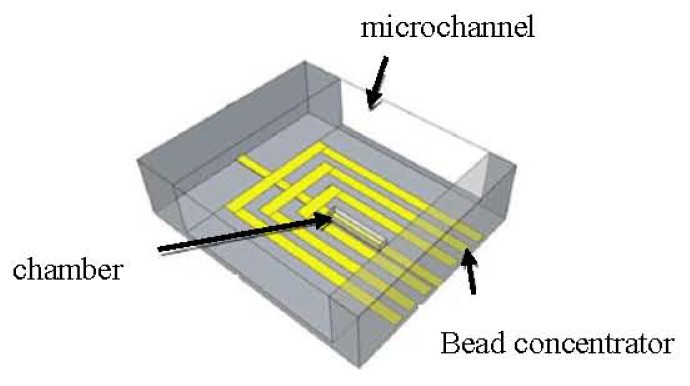
Schematic of the device consisting of the microchannel, the bead concentrator, and the chamber with gold microstructure underneath [34].

As shown in Figure 7, the device comprises a bead concentrator consisting of gold microstructures at the bottom of a microchannel, which was fabricated by a standard photolithography process and 100 μm thin SU8 layer. The bead concentrator is used to attract and move MNPs into a trap at the center [33,76]. The trap is made of an SU8 chamber, 5 μm thickness, with a gold microstructure underneath and is used to attract and immobilize a defined number of MNPs. If a sample solution contains only MNPs but no bioanalyte, the number of particles that are trapped inside the chamber will be larger than in case the target is present in the sample solution. This is due to the increase in size as the targets bind to MNPs, which will cause the chamber to fill up with a smaller number of particles. Therefore, the difference in volume between the bare MNPs and the compound of MNPs and the bioanalyte in combination with the trap is the basis for the proposed detection method. In order to maintain a high sensitivity, the size of the target analyte and the MNP should be in the same range.

**Figure 8:**
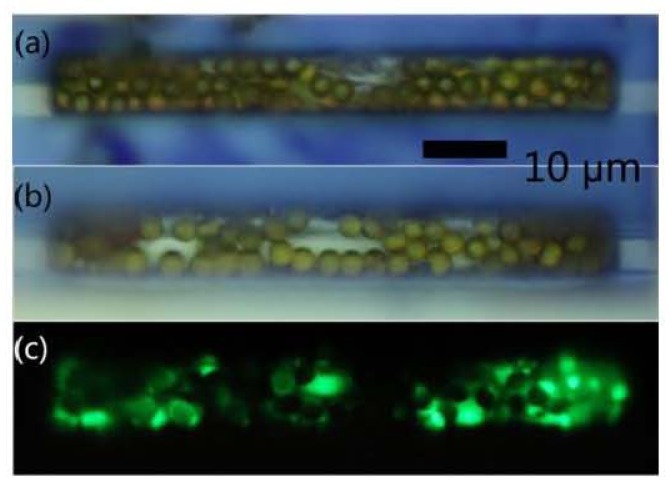
(**a**) About 60 magnetic particles trapped inside the chamber; (**b**) About 44 magnetic particles with BSA and fluorescent markers trapped; (**c**) Fluorescent markers in the trap [81].

A decrease of ~36% in the number of trapped MNPs was measured in experiments that were conducted using 2.8 μm streptavidin-coated Dynabead M-270 MNPs attached to 1 μm fluorescent particles (Fluoresbrite YG Carboxylate Microspheres) through Bovine Serum Albumin (BSA) protein as shown in Figure 8 [81].

TMR sensors are integrated into the system to enable the MNP detection and counting. The advantage is that complex biological treatment of the sensor surface is not required, since a combination of magnetic forces and a mechanical trap is used to immobilize the MNPs. The magnetic field generated by electrical currents can also be used to magnetize the MNPs. This eliminates the need for an external magnetic field source, which is commonly required for magnetoresistive biosensors. An aspect worth mentioning is that the concentrator would allow this system to operate on droplets rather than using microfluidic channels, which would reduce the complexity.

The detection of single micrometer size MNPs, which move quickly (cm s^−1^ velocities) through microfluidic channels was demonstrated using MR sensors integrated on the channel bottom [82,83] but the work presented in [84] was actually the first demonstration of this technique for cytometer applications. The authors proved that the suggested detection system can be used as a flow chip cytometer having the same efficiency, for high concentration samples, of the hemocytometer method and lesser error (4.5% in comparison to 8.5%). They managed to detect Kg1-a cells labeled with 50 nm MNPs in rapid flow (cm s^−1^), by spin-valve (SV) sensors, through a microchannel.

The microfabricated device was designed to incorporate 3 μm wide and 40 μm long SV sensors integrated to a 150 μm wide and 14 μm thick microfluidic channels, which were fabricated using polydimethylsiloxane (PDMS) and a micro-molding technique. The sensing elements of the device were three SV sensors, microfabricated on a Si (100) wafer passivated with 50 nm of Al_2_O_3_ deposited by RF magnetron sputtering. These SVs were top pinned sensors, fabricated by ion beam deposition and had the following structure as described in [84] substrate/Ta(2.0)/Ni_80_Fe_20_(2.5)/Co_80_Fe_20_(2.5)/Cu(2.0)/Co_80_Fe_20_(2.5)/Mn_76_Ir_24_(6.0)/Ta(2.0)/TiW(N2)(15) all thickness in nm. The MR signal was 7.69% with a sensitivity of 1.6% per mT, leading to a sensitivity of 4.8 V T^−1^ for a 1 mA bias current. The detection experiment was performed with cells diluted in phosphate buffer solution (100 mM, pH 7.4) in a concentration of 4.76 × 10^3^ cells/μL, flowing inside the microchannels with speeds around 1 cm s^−1^. The cell counts of 3, 6 μL sample obtained with the MR chip successfully led to an average measured number of 14,037 cells.

Another very interesting and novel method for in-flow detection of MNPs was recently reported in [85]. The researchers of [85] produced an elastic and stretchable GMR sensor and wrapped it around capillary tubing. Thus, the stray fields induced by the flowing MNPs could be detected virtually in all directions (isotropic sensitivity); a unique feature for elastic sensors when compared to their rigid planar counterparts. As mentioned previously [30,44,47,72,86] the detection of MNPs in fluidic flow requires high sensitivity of the sensor to magnetic fields in the range of 1 mT and below. Therefore the group focused on maximizing the sensitivity of their sensor on elastomeric membrane. They fabricated different GMR multilayer systems, including Co/Cu and Py/Cu stacks (Py = Ni_81_Fe_19_), on a free-standing rubber membrane. In all cases the GMR systems reveal a similar GMR performance on the poly(dimethylsiloxane) (PDMS) membrane and on rigid SiO_x_ wafers. In their paper they demonstrated that the sensitivity of the GMR sensor was remarkably high 106 T^−1^, with a maximum at a field of 0.8 mT. According to [85], the sensitivity of the sensor element was defined as the first derivative of the sample’s resistance over the magnetic field divided by the resistance value: S(*H*_ext_) = [d*R*(*H*_ext_)/d *H*_ext_]/*R*(*H*_ext_). The obtained GMR curve was narrow with a very low saturation field and a considerable resistance change of more than 13%.

Finally, in [87], researchers from the University of Colorado at Boulder and the National Institute of Standards and Technology in USA presented a novel microfluidic platform to trap, release, transport, and detect MNPs with SVs. The reported technique for simultaneous MNP manipulation and detection was demonstrated on a linear array of bistable 1 μm × 8 μm bottom-pin SVs (~3% GMR ratio) addressed locally with read and write lines. In particular, the array consisted of two staggered lines of 1 μm × 8 μm SVs turned ON or OFF with current pulsed through 150 nm thick and 8 μm wide write lines.

The bottom-pin SV consisted of (thicknesses in nanometers): Ta(3)/Cu(3)/Ir_20_Mn_80_(10)/ Ni_80_Fe_20_(15)/Co_90_Fe_10_(5)/Cu(10)/Co_90_Fe_10_(5)/Ni_80_Fe_20_(15)/Ta(5)). A PDMS microfluidic chip was bonded to the surface of the SV chip. A low concentration (<100 beads/mL) of streptavidin-functionalized 2.8 μm diameter MNPs was injected into the 40 μm deep and 80 μm wide channel by means of a micro-fluidic port (MFP) and syringe pump (0.001–0.005 μL/min). Computer-controlled lock-in amplifiers and current sources enabled automation of the SV SPB transport and detection processes. It was shown that the bistable SV could simultaneously capture and detect a single 2.8 μm MNP and a linear array of these SVs could precisely transport the MNPs. One of the advantages of this method is that the MNPs are not permanently immobilized and there is low heat production due to short current pulses.

## 3. Conclusions

In this paper, we reviewed some of the most recent results in research towards microfluidic biosensing using MR sensors and magnetic particles. The presented biosensing systems are very promising candidates for lab-on-a-chip devices as compact, ultra-sensitive and inexpensive solutions for clinical diagnostics and biomedical applications in general. Our study outlined that several novel manipulation, separation and detection mechanisms based on magnetic methods are continuously emerging, proving that magnetic biosensing has the potential to become competitive and probably replace in the future the current optical and fluorescence detection technologies, while maintaining the high sensitivity and fast readout time. Compared to these methods, the magnetic microfluidic biosensors measure directly the electrical signal from the MR sensor, provide a fully electronic readout and thus enable the development of portable, hand-held devices. Fluorescence systems suffer from bleaching, spectral overlapping and the detection optics are associated with higher costs [88]. Extensive mathematical corrections and calibration are necessary for a successful detection. On the contrary, magnetic labeling of bioanalyte and detection by MR based sensors do not suffer from such constrains. By combing these systems with on-chip magnetic field generators as mentioned in [33,34,76–81] for rapid, selective manipulation and trapping of MNPs at the sensor site, the detection time is reduced since there is no need for hybridization on the sensor’s surface as in other detection processes.

In recent years, a lot of progress was made in optimizing the device performance; single magnetic nanoparticle detection has been achieved using TMR sensors opening the way for single molecule detection applications. This approach with microfluidics, MNPs and MR based sensors offers a stable labeling system using low-cost components. Magnetic biosensing could complement, or even replace in the near future, the existing fluorescence based biosensing methods since it facilitates manipulation, detection and sorting of bioanalyte on a single chip.
